# Global and regional degree of obstruction determined by electrical impedance tomography in patients with obstructive ventilatory defect

**DOI:** 10.1371/journal.pone.0209473

**Published:** 2018-12-20

**Authors:** Chao Zhang, Meng Dai, Wei Liu, Xiaohui Bai, Jiaming Wu, Canhua Xu, Junying Xia, Feng Fu, Xuetao Shi, Xiuzhen Dong, Faguang Jin, Fusheng You

**Affiliations:** 1 Department of Biomedical Engineering, Fourth Military Medical University, Xi’an, Shaanxi, China; 2 Medical Engineering Section, General Hospital of Shenyang Military Region, Shenyang, Liaoning, China; 3 Department of respiratory medicine, Tangdu Hospital, Fourth Military Medical University, Xi’an, Shaanxi, China; 4 The Fifth People’s Hospital of Baoji City, Baoji, Shaanxi, China; 5 Department of Medical Technology, Bethune Military Medical NCO Academy of PLA, Shijiazhuang, Hebei, China; University of Pittsburgh, UNITED STATES

## Abstract

**Background:**

Electrical impedance tomography is a continuous imaging method capable of measuring lung volume changes. The purpose of this study was to examine whether EIT was capable of evaluating the degree of obstructive ventilatory defect (OVD) on the global and regional level.

**Methods:**

41 healthy subjects with no lung diseases and 67 subjects suffering from obstructive lung diseases were examined using EIT and spirometry during forced vital capacity (FVC) maneuver. The subjects were divided into control group (n = 41), early airway obstruction group (n = 26), mild group (n = 17), moderate group (n = 16) and severe group (n = 8) according to the degree of obstruction. Forced expiratory volume in 1 second (FEV_1_) and FEV_1_/FVC were determined by EIT. The mode index (MI) was proposed to evaluate the degree of global and regional obstruction; the effectiveness of MI was validated by evaluating posture related change of lung emptying capacity in sitting and supine postures; the degree of regional obstruction was determined according to the cut-off values of MI obtained from receiver operating characteristic (ROC) analysis; regional obstruction was located in the four-quadrant region of interest (ROI) and the contour-map ROI with contour lines at the cut-off values of MI.

**Results:**

Significant differences were found between different groups (P<0.05) and the global MI was 0.93±0.03, 0.86±0.05, 0.81±0.09, 0.73±0.09 and 0.60±0.11 (mean ±SD), respectively. The cut-off MI value was 0.90, 0.83, 0.77, and 0.65, respectively.

**Conclusion:**

The results indicated the potential of EIT to evaluate the degree of obstruction in patients with obstructive ventilatory defect on the global and regional level.

## Introduction

Obstructive lung diseases such as bronchial asthma and chronic obstructive pulmonary disease (COPD) often have in common an obstructive ventilatory defect (OVD), though having different pathogenesis. OVD is defined by a reduced FEV1/VC ratio below the 5th percentile of the predicted value [1], leading to an inhomogeneous ventilation distribution. Clinically, OVD is identified using pulmonary function tests (PFT). While PFT provides only global parameters representing the disease state of the entire lung without regional information on inhomogeneous ventilation. A continuous and bedside evaluation both on the global and regional ventilation could be more helpful to estimate the progression and therapy effects of obstructive lung diseases.

Electrical impedance tomography (EIT) is a portable, noninvasive, and radiation-free functional imaging method [2], which produces the electrical impedance image by injecting safe currents of certain frequency and then measuring changes in surface voltages via electrodes placed on the circumference of chest [3,4,5]. Regional ventilation-related changes in lung aeration modulate regional electrical current and the resulting changes in electrical lung tissue impedance are proportional to regional changes in gas content [6], thus the inhomogeneity of ventilation distribution can be measured by EIT. Besides, the safety, low cost, and high temporal resolution of EIT make it a promising tool to monitor pulmonary ventilation non-invasively and dynamically [7,8].

The advantages of EIT have driven it toward clinical application. In the clinical setting, EIT is used to monitor the mechanical ventilation of critically ill patients. EIT assists physicians to set ventilator parameters by monitoring and evaluating the ventilation distribution continuously [9,10,11,12,13], securing the lung-protective ventilator setting [14,15]. EIT examinations of diseases such as pneumothorax [16], cystic fibrosis [17,18], and obstructive sleep apnea syndrome [19] have been documented.

Evaluation on obstructive lung diseases is a major focus of EIT research. In order to analyze the effect of inhaled bronchodilator on the regional level, EIT and PFT were applied in COPD and asthma patients before and after bronchodilator reversibility testing [20,21]. Heterogeneity of regional lung ventilation was compared using EIT on young and elderly subjects without lung disease and COPD patients [6]. The results of these studies showed the potential of EIT to monitor the inhomogeneity of ventilation distribution in patients with obstructive lung diseases, while the degree of obstruction was not discussed. Meanwhile, our initial observations in patients with obstructive lung disease indicated that the histogram of pixel FEV1/FVC value changed with the degree of obstruction. We thus realized that EIT might be a potential method to detect and evaluate both global and regional degree of OVD.

Therefore, the aim of this study was to determine whether EIT was able to evaluate the degree of obstruction in patients with different degrees of OVD on the global and regional level. In order to achieve this goal, forced vital capacity (FVC) was measured using EIT and spirometry on patients with different degrees of OVD. Forced expiratory volume in 1 second (FEV_1_) and the FEV_1_/FVC ratio were determined in EIT images; an evaluation method was proposed to evaluate the degree of global and regional obstruction.

## Materials and methods

### Ethics statement

The study involved human participants and was approved by the Ethics Committee of the Fourth Military Medical University (approval number: FMMU-E-III-001(1–7)). Written informed consent was obtained from all subjects. The whole procedures were conducted according to the Principles of Helsinki.

### Subjects

108 Subjects were recruited from respiratory outpatient clinic of Tangdu hospital (Xi’an, China) between March 2015 and September 2016. Exclusion criteria included: age<18years; pregnancy or lactation; contraindications to PFT; and any EIT contraindications (e.g., pacemakers, implantable defibrillators). The subjects involved: (1) 41 healthy subjects with no history of lung disease and smoking as a control group; (2) 26 subjects who were diagnosed as early airway obstruction in PFT; (3) 41 subjects who were diagnosed as OVD in PFT, including 24 outpatients with COPD and 17 outpatients with asthma attacks. Regarding personal privacy, all individually identifiable health information (e.g., personal identification or hospital identification number) has been encrypted prior to release.

The degree of obstruction was determined according to the ATS/ERS guidelines which recommend using FEV_1_%pred as the metric to define severity of obstructive ventilatory defect [1]. Predicted values and lower limit of normal (LLN) for lung function were obtained using the Global Lung Function Initiative (GLI) 2012 equations of the North East Asian ethnic group [22]. Subjects were divided into 5 groups:

1. Control group (n = 41, healthy subjects);
2. Early airway obstruction group (n = 26, normal FEV_1_ and FEV_1_/FVC, but Forced expiratory flow over the middle half of the FVC (FEF_25-75%_) <65% of predicted) [23];
3. Mild group (n = 17, FEV_1_/FVC < LLN & 70% ≤ FEV_1_%pred < LLN);
4. Moderate group (n = 16, FEV_1_/ FVC < LLN & 50% ≤ FEV_1_%pred < 70%);
5. Severe group (n = 8, FEV_1_/FVC < LLN & FEV_1_%pred < 50%).

Anthropometric and spirometric data of subjects are given in Table 1.

**Table 1:** Anthropometric and spirometric data of the subjects in 5 groups.

Variable	Control (n = 41)	Early (n = 26)	Mild (n = 17)	Moderate (n = 16)	Severe (n = 8)
**Age(years)**	45.02±12.86	45.54±14.17	48.41±12.69	51.19±11.75	55.75±8.42
**Height(cm)**	164.15±7.96	163.35±8.30	163.29±5.62	166.50±6.19	167.87±4.61
**Weight(kg)**	67.05±10.51	63.96±10.57	64.65±12.56	63.62±10.56	71.87±5.59
**FEV**_**1**_**%pred.**	95.68±9.55	88.51±6.05	74.17±2.52	58.33±5.46	34.08±8.73
**FVC %pred.**	90.33±7.60	87.35±4.22	87.14±3.03	70.52±6.17	48.36±9.23
**FEV**_**1**_**%FVC**	86.51±5.72	82.86±4.45	69.29±4.27	66.51±4.28	56.01±8.88
**FEF**_**25-75%**_**%pred**	87.70±18.25	58.89±6.28	55.97±10.45	40.69±9.81	21.23±11.91

Data are expressed as mean ±SD. FEV1, forced expiratory volume in 1 second; FVC, forced vital capacity; FEF_25-75%_, forced expiratory flow at 25 and 75% of the pulmonary volume; %pred. = %predict.

### EIT data acquisition and analysis

**Fig 1:**
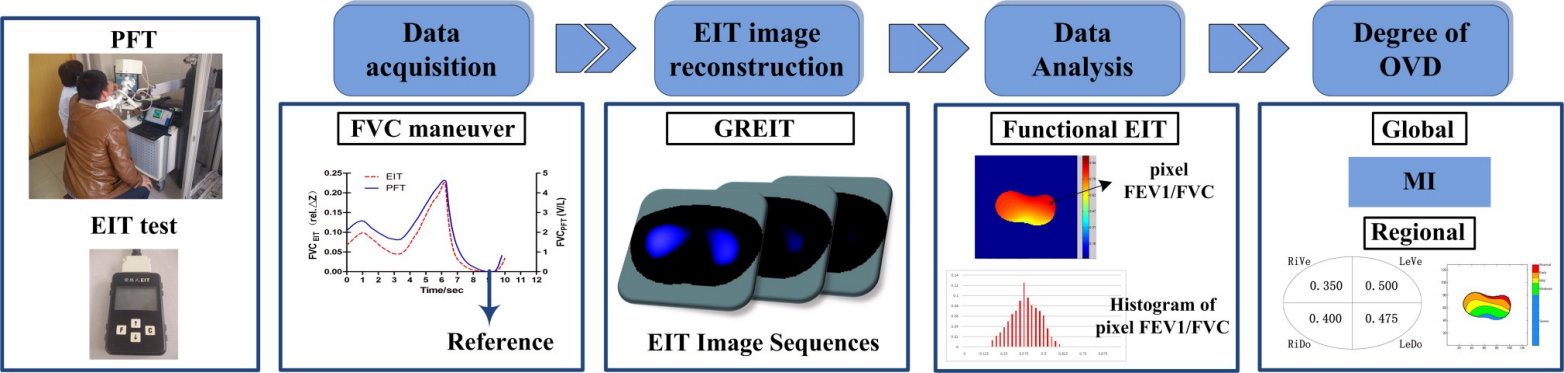
Sequence of processes involved in EIT examination and data analysis. The patient has given written informed consent, as outlined in the PLOS consent form, to publication of his photography.

Sequence of processes involved in EIT examination and data analysis was shown in Fig 1.

### EIT data acquisition

Sixteen electrodes (Disposable ECG electrode YH-1Ag/AgCl, Tianrun Sunshine Medical Supplies Co., Ltd, Beijing, People’s Republic of China) were placed on the chest circumference in the 5–6th intercostal space [20,24]. All subjects were examined by spirometry (Jaeger Pneumotachograph, CareFusion GmbH, Höchberg, Germany) and EIT (PEIT4 system, Fourth Military Medical University, Xi’an, People’s Republic of China) simultaneously in a seated position during tidal breathing and subsequent full inspiration from residual volume to total lung capacity followed by standard forced full expiration maneuver. The examinations were performed before the bronchial dilation test or bronchial provocation test.

EIT data were recorded in parallel with spirometry using the PEIT4 system. Unlike traditional EIT system that uses adjacent pattern of current injection and voltage measurement and results in 208 measurements per frame [6], PEIT4 is a 16-electrode EIT system that uses the opposite pattern of electrical current drive [25] and adjacent pattern of voltage measurement. During each measuring cycle, 16 consecutive current injections were performed. The resulting voltages were measured with 12 passive adjacent electrode pairs during each of these current injections (the electrode pairs including active electrodes were excluded from voltage measurement). One hundred and ninety-two (16×12) voltages were acquired during each cycle. The voltage measurement accuracy of PEIT4 is better than 1‰, the electrical excitation current is of 1mA and 100 kHz, and the EIT images were acquired at a scan rate of 12 scans /s.

### EIT image reconstruction

Further EIT data analysis was performed offline. EIT images (each consisting of 128×128 pixels) were generated from the raw data acquired in each cycle using the GREIT software with EIDORS version 3.9 [26], and the reconstruction was performed using the adult human thorax model geometry predefined in EIDORS. The pixel impedance after full expiration was chosen as the reference pixel impedance (Z_ref_), and the pixels in EIT images showed the difference between the instantaneous pixel impedance Z and the reference pixel impedance Z_ref_ normalized by Z_ref_, i.e., (Z-Z_ref_)/Z_ref_. This value is known as the relative impedance change (rel.ΔZ) [6]. Further details about EIT image reconstruction can be found elsewhere [27,28].

The lung area of an EIT image was defined, pixel values larger than 20% of the maximum pixel FVC value were marked as identified lung area. Subsequent data analysis was based on the lung area. Functional EIT (fEIT) images were calculated to show the airway resistance to airflow, in which the pixel values showed the ratio of pixel FEV_1_ to pixel FVC, corresponding to the FEV_1_/FVC in PFT.

### Calculation of EIT index

To evaluate the degree of OVD, the distribution of pixel FEV_1_/FVC values in the lung area or in the region of interest (ROI) was presented as histogram of 40 bins. The airflow measured by spirometry is influenced by the resistance of the respiratory tract, and the resistance change caused by obstructive lung diseases leads to a change in the distribution of pixel FEV1/FVC measured by EIT. Therefore, the mode index (MI), reflecting the pixel FEV_1_/FVC value that appeared most often, was chosen to quantify the degree of global and regional obstruction:

$$p(r_{k})=n_{k}/n\;\left(k=1,2,3,\ldots 40\right)$$ (1)

$$MI=m/40\;when,p(r_{m})=\max(p(r_{k}))$$ (2)

Where, p(r_k_) is the normalized frequency of bin k; n_k_ is the number of pixels in bin k; n is the total number of all pixels. m is the number of the bin that has the largest frequency.

### Effectiveness validation of the MI on healthy subjects

To verify the capacity of the proposed MI evaluating the global and regional ventilation, 15 healthy adults were tested in the sitting and supine postures during forced vital capacity maneuver. The effectiveness of MI evaluating disease related airflow limitation was validated by evaluating body position related change of lung emptying capacity [29, 30].

The lung area in a fEIT image was divided into four quadrants [18]: right ventral (RiVe), left ventral (LeVe), right dorsal (RiDo), and left dorsal (LeDo). EIT images, fEIT images and the regional MIs in the four quadrants were obtained in the sitting and supine postures.

### Evaluation on the global degree of obstruction

To evaluate the global degree of OVD, the described analysis was identically performed using all the EIT data of subjects in the 5 groups. The frequency distributions of pixel FEV_1_/FVC values were calculated and presented as histograms. The global MIs of all subjects in different groups were presented as box graphs. Receiver operating characteristic (ROC) analysis was used to characterize the power of MI to discriminate the subjects with different degrees of OVD, the cut-off values of MI were obtained based on the biggest Youden's index.

### Evaluation on the regional degree of obstruction

A contour-map ROI was defined to locate the regional obstruction. The fEIT images were presented as a contour map with contour lines at the specified pixel FEV_1_/FVC values equaling to the cut-off values of MI. The regions separated by the contour lines were different ROIs with different degrees of obstruction. Regional obstruction was located to the contour-map and the four-quadrant ROIs. The degree of regional obstruction in the four-quadrant ROI was determined by comparing the regional MI to the cut-off values of MI.

### Statistical analysis

Statistical analysis was performed with GraphPad Prism version 5 for Windows (GraphPad Software, Inc., La Jolla, CA, USA). One-way analysis of variance with Bonferroni post test was used to test the significance of differences in MIs of different groups, P<0.05 were deemed significant. The significance of differences in the proportion of regional degree in four-quadrant ROIs of each group was tested with Chi-square test or Fisher's exact test. P <0.005 were deemed significant according to Bonferroni correction method.

## Results

### Gravity-dependent redistribution of ventilation evaluated by MI

**Fig 2:**
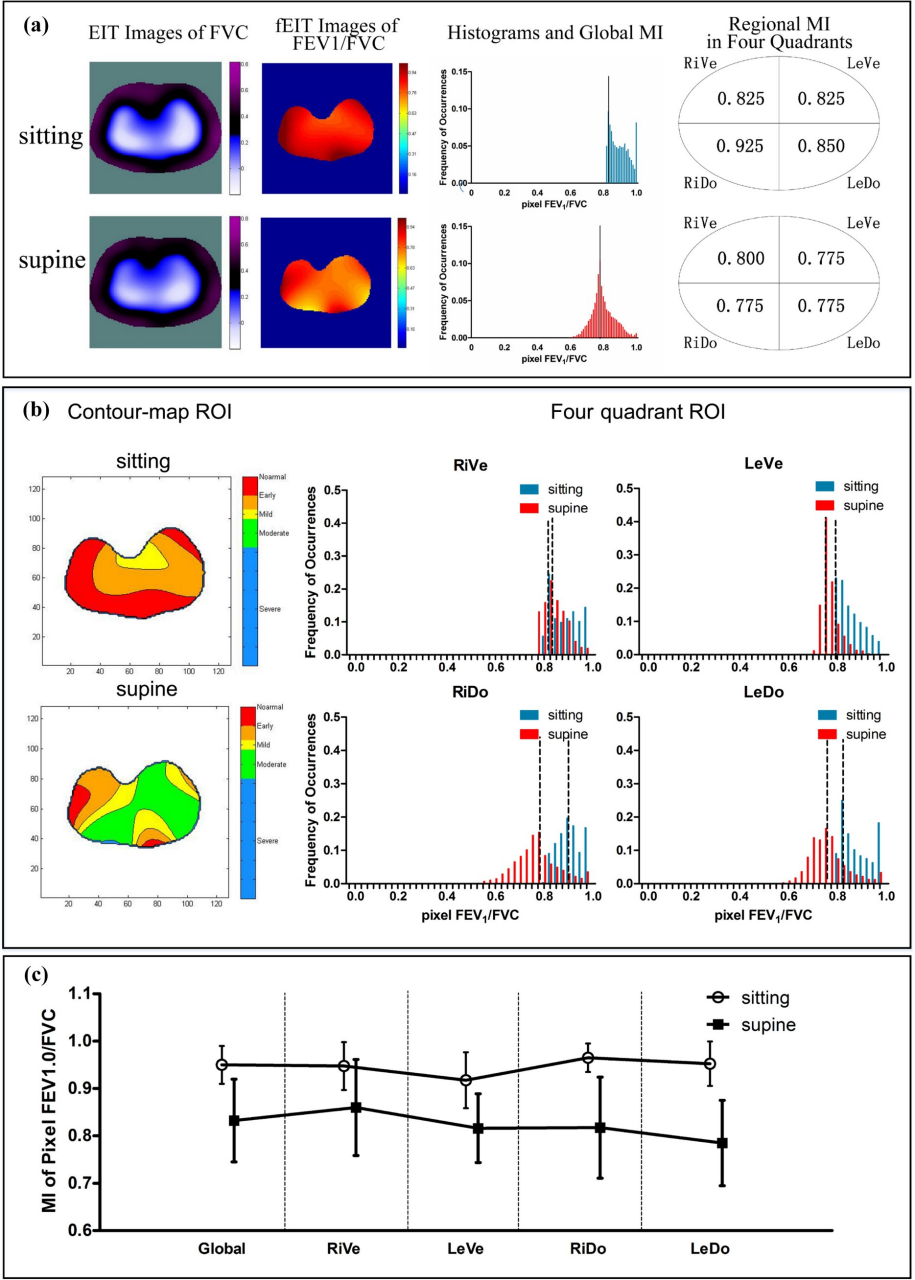
The distribution of ventilation evaluated by EIT in different postures. (a) EIT images of FVC, fEIT images of pixel FEV1/FVC, Histograms and global MI, and regional MIs in four quadrants obtained in a healthy subject in sitting (top) and supine (bottom) posture. (b) Regional obstruction in the contour-map ROI and the changes of the histogram in the four-quadrant ROIs in sitting and supine postures. Dashed lines show the global and regional MIs. (c) Global and regional MIs (mean±SD) of all healthy subjects in sitting and supine postures. The image orientation is the following: ventral is at the bottom and the right side of the body is on the left of the image. FEV1, forced expiratory volume in 1 second; FVC, forced vital capacity; RiVe, right ventral; LeVe, left ventral; RiDo, right dorsal; LeDo, left dorsal.

Fig 2(A) shows the EIT images, fEIT images, histograms and global MI, and the regional MI in four quadrants of one healthy subject in sitting and supine posture, respectively. The EIT images of the two postures showed a decreased impedance and a redistribution of regional ventilation, the pixel FEV_1_/FVC values of fEIT images decreased in the supine posture. The global and regional MIs showed the difference in the distribution of pixel FEV_1_/FVC between different postures. Fig 2(B) shows the regional obstruction in the contour-map ROIs and the changes of the histogram in the four-quadrant ROIs in sitting and supine postures. Fig 2(C) shows the global and regional MIs in four quadrants of all the tested subjects in sitting and supine postures.

### Global degree of obstruction evaluated by EIT

**Fig 3:**
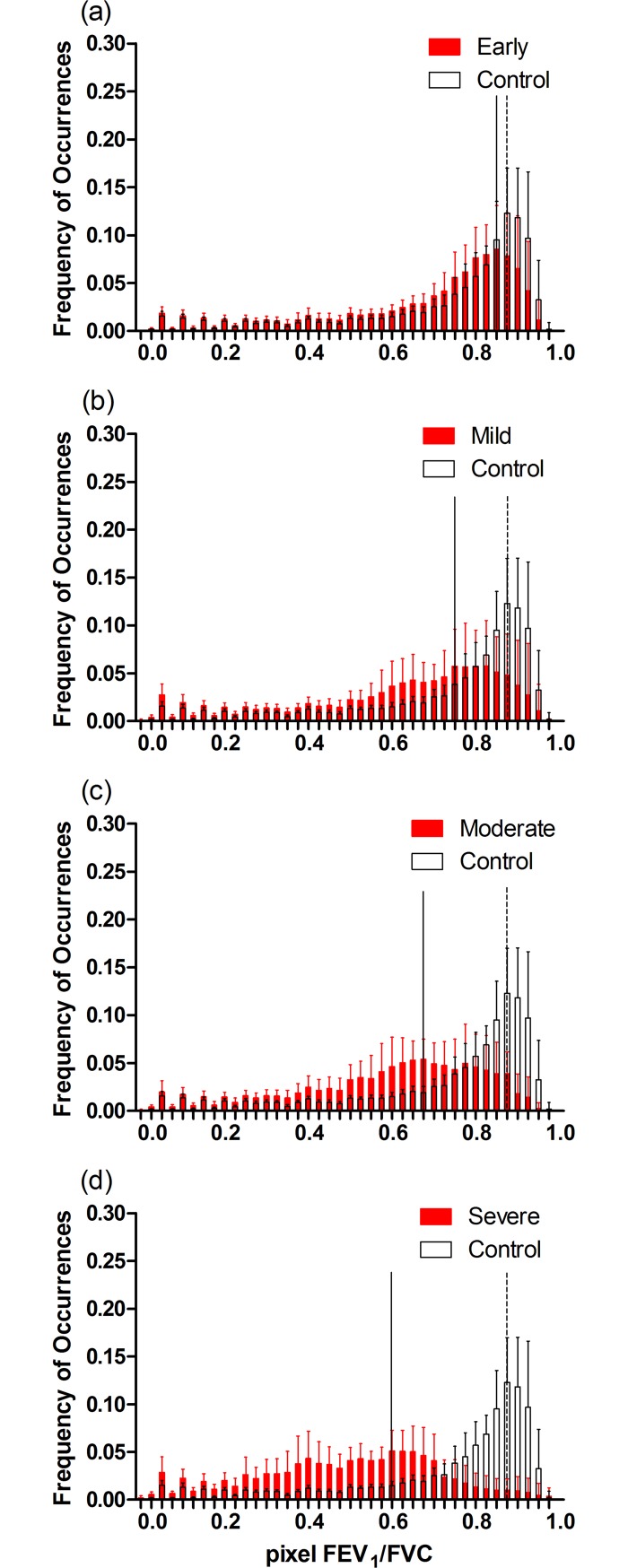
Histograms of pixel FEV_1_/FVC in the 5 groups. Histograms of the groups with obstruction are shown in red solid columns in comparison with the control group (open columns). (a) Early airway obstruction group and Control group; (b) Mild group and Control group; (c) Moderate group and Control group; (d) Severe group and Control group. Values are presented as mean ± SD. Solid and dashed lines show the MIs in the experimental and control groups, respectively. FEV1, forced expiratory volume in 1 second; FVC, forced vital capacity.

The histograms of pixel FEV_1_/FVC values show the differences in the distribution of ventilation between the control group and the groups with different degrees of obstruction (Fig 3). As expected, the peak of histogram in the control group is closer to 1 than those in the other groups. As the degree of obstruction aggravated, the peaks of the ventilation distribution showed a left-shift trend towards 0. The more severe the obstruction, the more dispersed and the less homogeneous the distribution of ventilation was.

**Fig 4:**
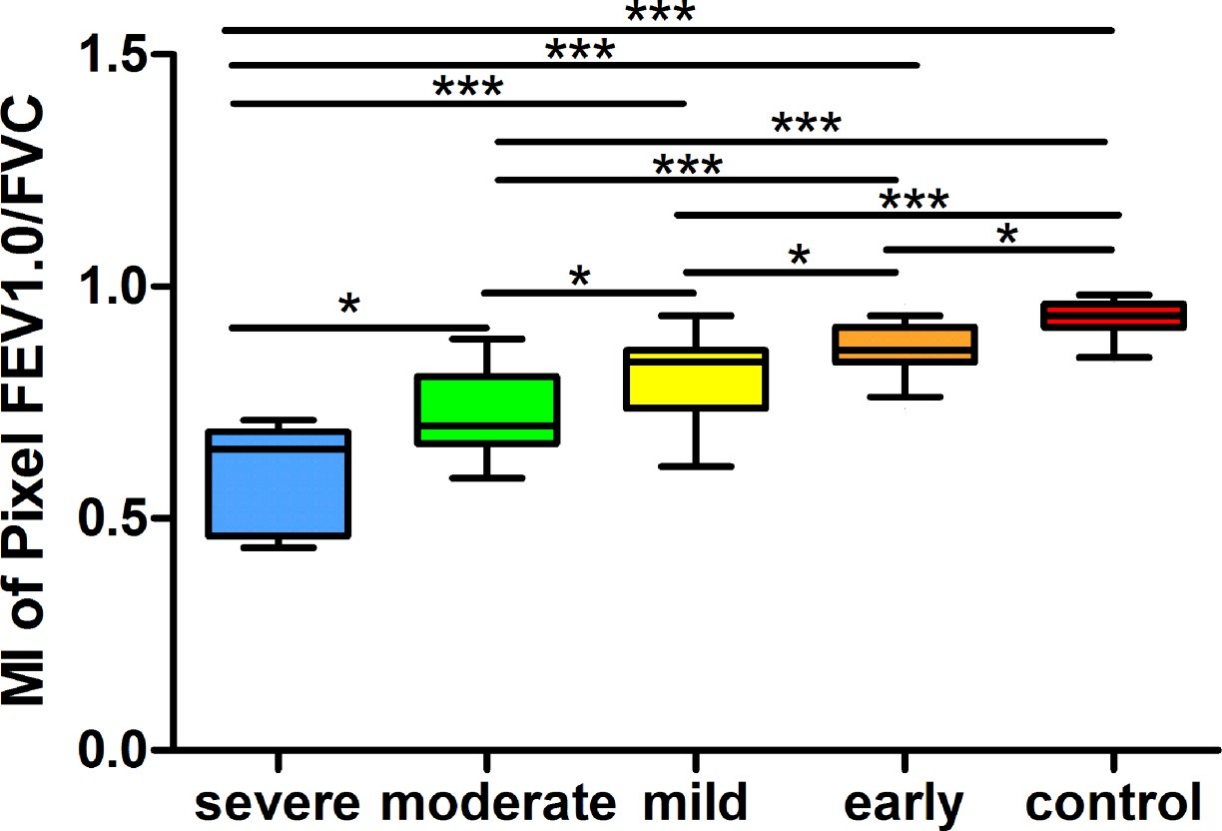
The MIs of pixel FEV1/FVC determined by EIT in the 5 groups. Boxes and whisker plots show the minimum, 25% percentile, median, 75% percentile, and maximum values. Significant differences among the groups are indicated by *p <0.05, **p<0.01, and ***p <0.005. EIT, electrical impedance tomography; MI, the Mode index. FEV1, forced expiratory volume in 1 second; FVC, forced vital capacity.

Fig 4 shows the differences in MIs between the groups with different degrees of obstruction. Consistent with the histograms in Fig 3, the MI of pixel FEV_1_/FVC values increased gradually with the alleviation of obstruction, the MI of different group was 0.54±0.11, 0.72±0.08, 0.80±0.06, 0.86±0.05, and 0.92±0.04 (mean ±SD), respectively. Significant differences were found in the MIs of all the groups.

**Fig 5:**
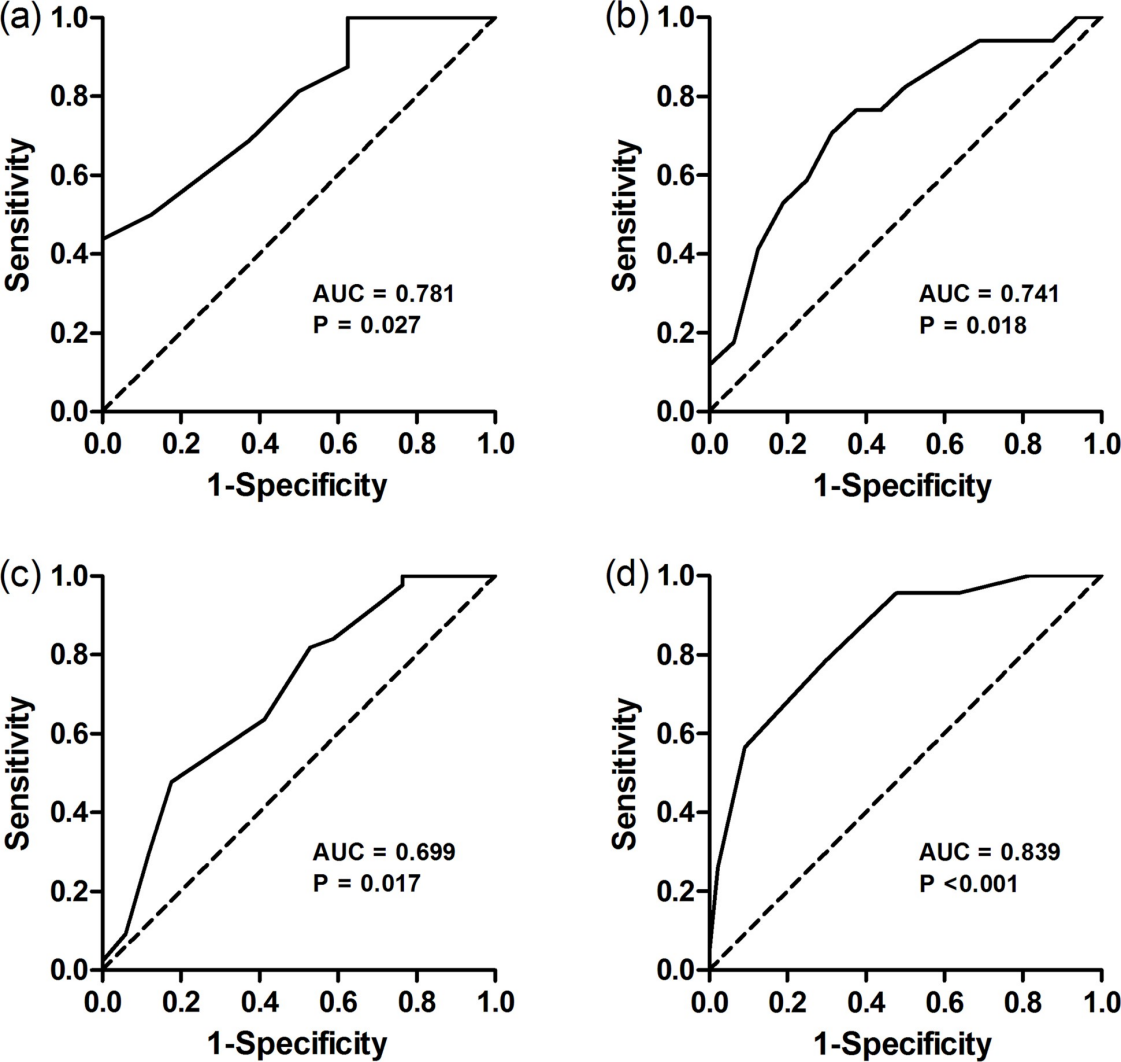
ROC curves indicating the power of MI to discriminate between different degrees of obstruction. (a) Severe and Moderate groups; (b) Moderate and Mild groups; (c) Mild and Early airway obstruction groups; (d) Early airway obstruction and Control groups. ROC curves (solid lines), line of identity (dashed line) and p values are given in each diagram. AUC, area under the curve.

ROC analysis was used to assess the power of MI in discriminating subjects with different degrees of obstruction (Fig 5). The area under the ROC curve of Severe and Moderate groups, Moderate and Mild groups, Mild and Early airway obstruction groups, Early airway obstruction and Control groups was 0.781, 0.741, 0.699 and 0.839(p<0.05), and the cut-off values was 0.650, 0.775, 0.825 and 0.9, respectively.

### Regional degree of obstruction evaluated by EIT

**Fig 6:**
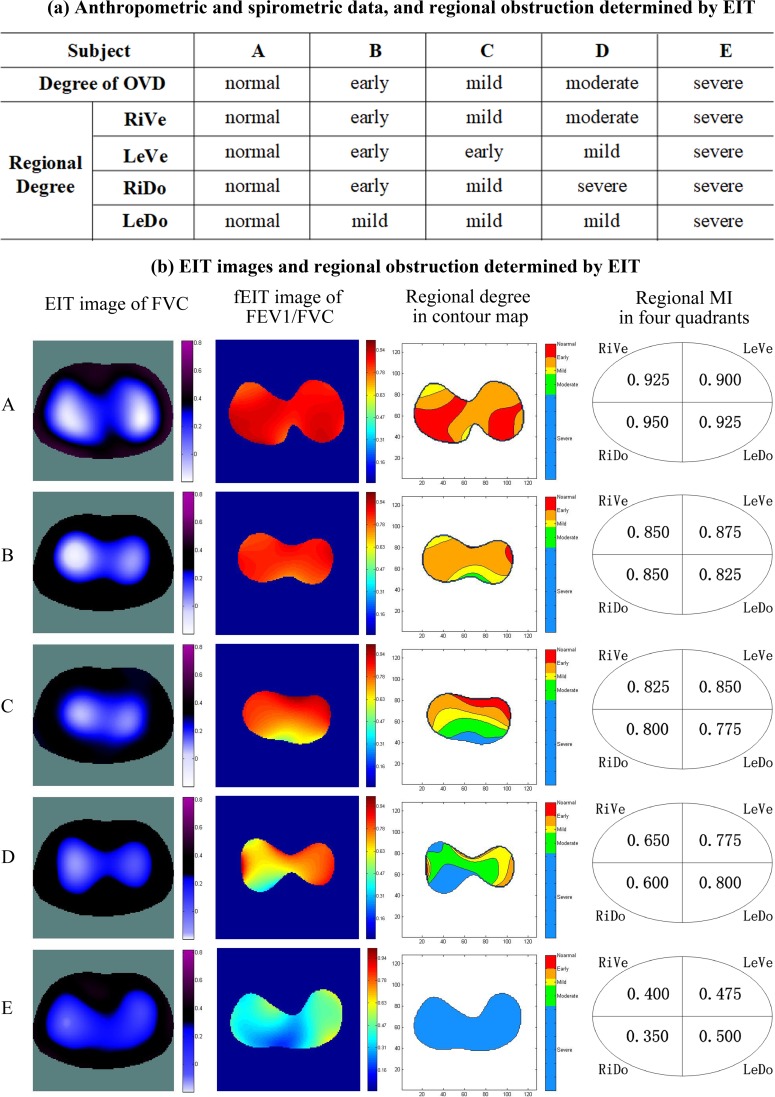
Anthropometric and spirometric data, EIT images and regional obstruction determined by EIT of 5 subjects. The image orientation is the following: ventral is at the bottom and the right side of the body is on the left of the image. FEV1, forced expiratory volume in 1 second; FVC, forced vital capacity. RiVe, right ventral; LeVe, left ventral; RiDo, right dorsal; LeDo, left dorsal.

Fig 6 shows the anthropometric and spirometric data, EIT images, and regional degree of obstruction determined by EIT of 5 subjects with different degrees of obstruction. Regional degree of obstruction in the four-quadrant ROI was determined by comparing the regional MI to the cut-off values of MI. Pixel values of fEIT images decreased and became inhomogeneous along with the degree of obstruction generally. The ROIs with different colors in the contour map marked different FEV_1_/FVC ranges, indicating different obstructive degrees. The area of each ROI varied along with the aggravation of obstructive degree.

**Fig 7:**
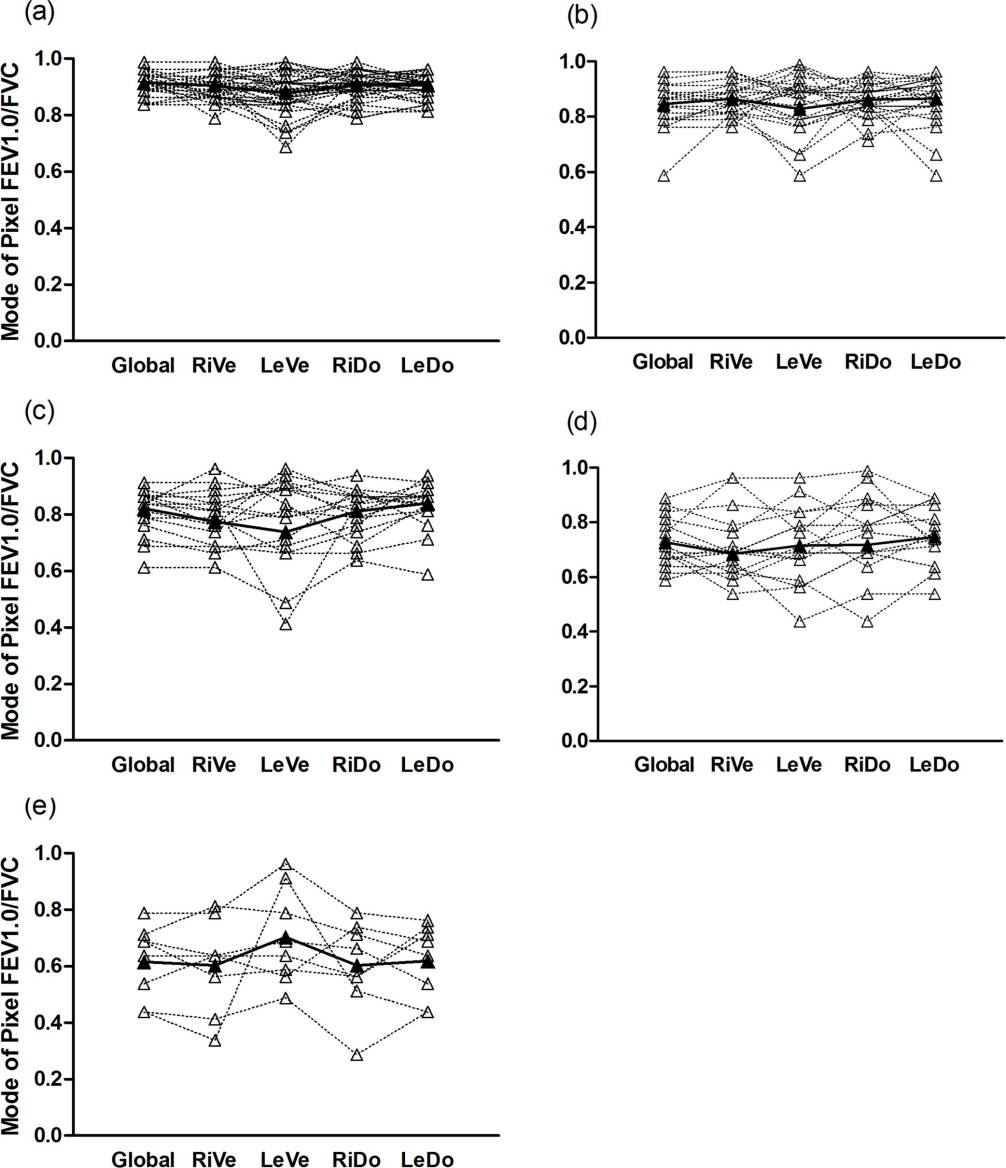
Global and regional MIs in the four quadrants of the 5 groups. (a) Control group. (b) Early airway obstruction group. (c) Mild group. (d) Moderate group. (e) Severe group. Individual (open symbols, dashed line) and mean values (solid symbols, solid lines) are shown. RiVe, right ventral; LeVe, left ventral; RiDo, right dorsal; LeDo, left dorsal; FEV1, forced expiratory volume in 1 second; FVC, forced vital capacity.

**Fig 8:**
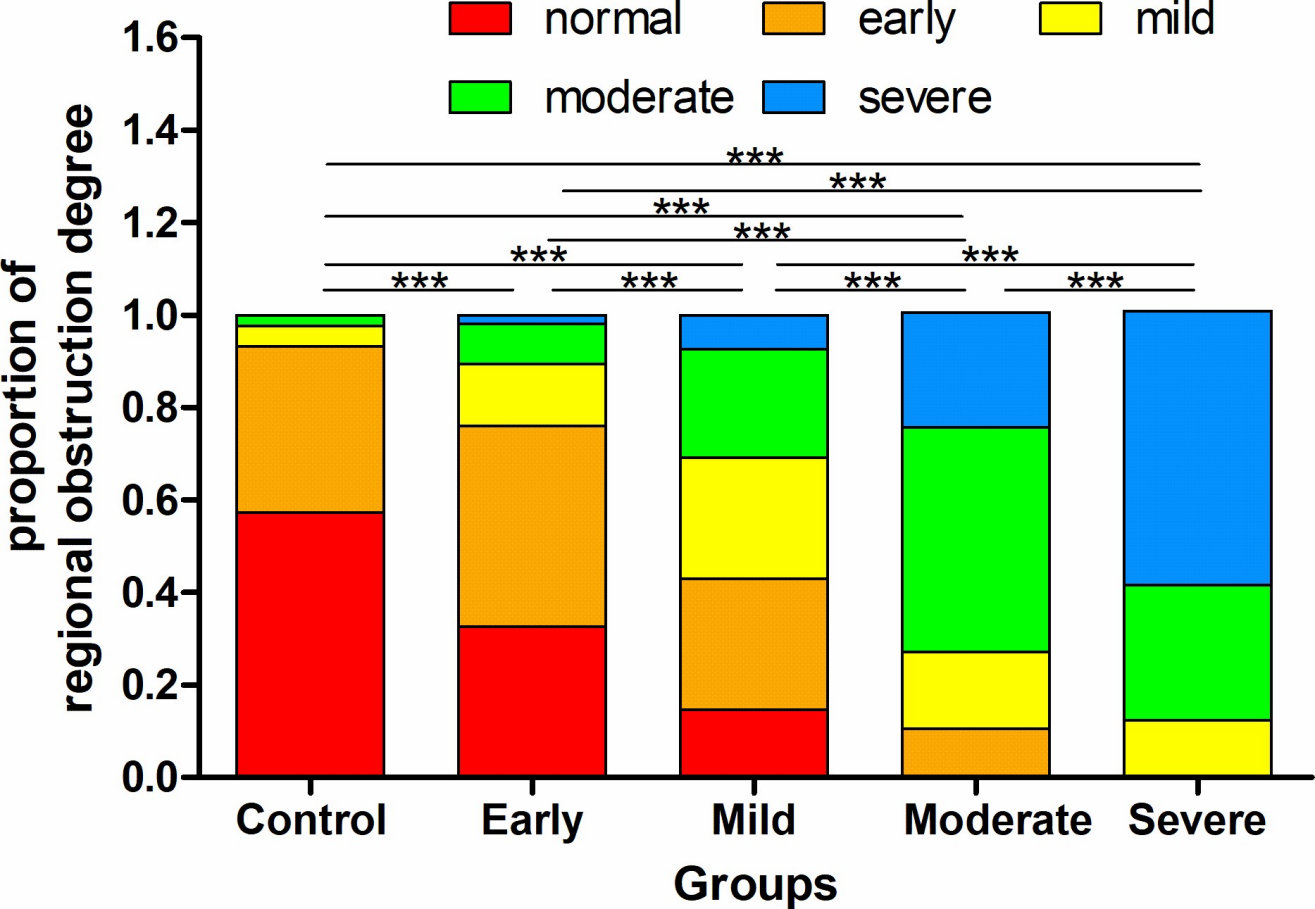
The proportion of regional degree in all the four-quadrant ROIs of each group. Significant differences among the groups are indicated by ***p <0.005.

Global and regional MIs in the four-quadrant ROIs show the individual differences of all the subjects in Fig 7. Both the global and regional MI decreased along with the aggravation of obstruction generally, but the regional MI might be larger or lower than the global MI, indicating a better or worse regional ventilation. Fig 8 shows the proportion of each regional degree determined by EIT in all the four-quadrant ROIs of each group.

## Discussion

In this study, subjects with obstructive lung diseases were examined using both EIT and spirometry to evaluate the degree of OVD. The MI was used to evaluate the global and regional degree of obstruction. The effectiveness of MI was validated by evaluating posture related change of lung emptying capacity in sitting and supine postures. The degree of regional obstruction was determined according to the cut-off values of MI obtained from receiver operating characteristic (ROC) analysis.

The differences between different degrees of OVD were assessed by counting the MI of pixel FEV_1_/FVC values in the fEIT image. The results showed the consistency between the MI of pixel FEV_1_/FVC determined by EIT and the severity of OVD determined by FEV_1_%pred in PFT. The MIs decreased and the distribution of pixel FEV_1_/FVC became scattered as the degree of obstruction aggravated, indicating a greater expiratory resistance and less homogeneous distribution of ventilation. The generated ROC curves showed the power of MI to discriminate between different degrees of obstruction, and the cut-off values of MI were obtained and used to evaluate the degree of regional obstruction.

### Effects of obstructive lung diseases on the ventilation distribution

Human lungs are not homogeneously ventilated, even under physiological conditions, because of posture and gravity [31]. Further heterogeneities in lung ventilation are introduced by lung disease, such as Asthma and COPD. Although both the two diseases are distinct conditions in terms of aetiological agent and reversibility of airway obstruction, they both cause OVD and share similar characteristics such as airway obstruction.

**Fig 9:**
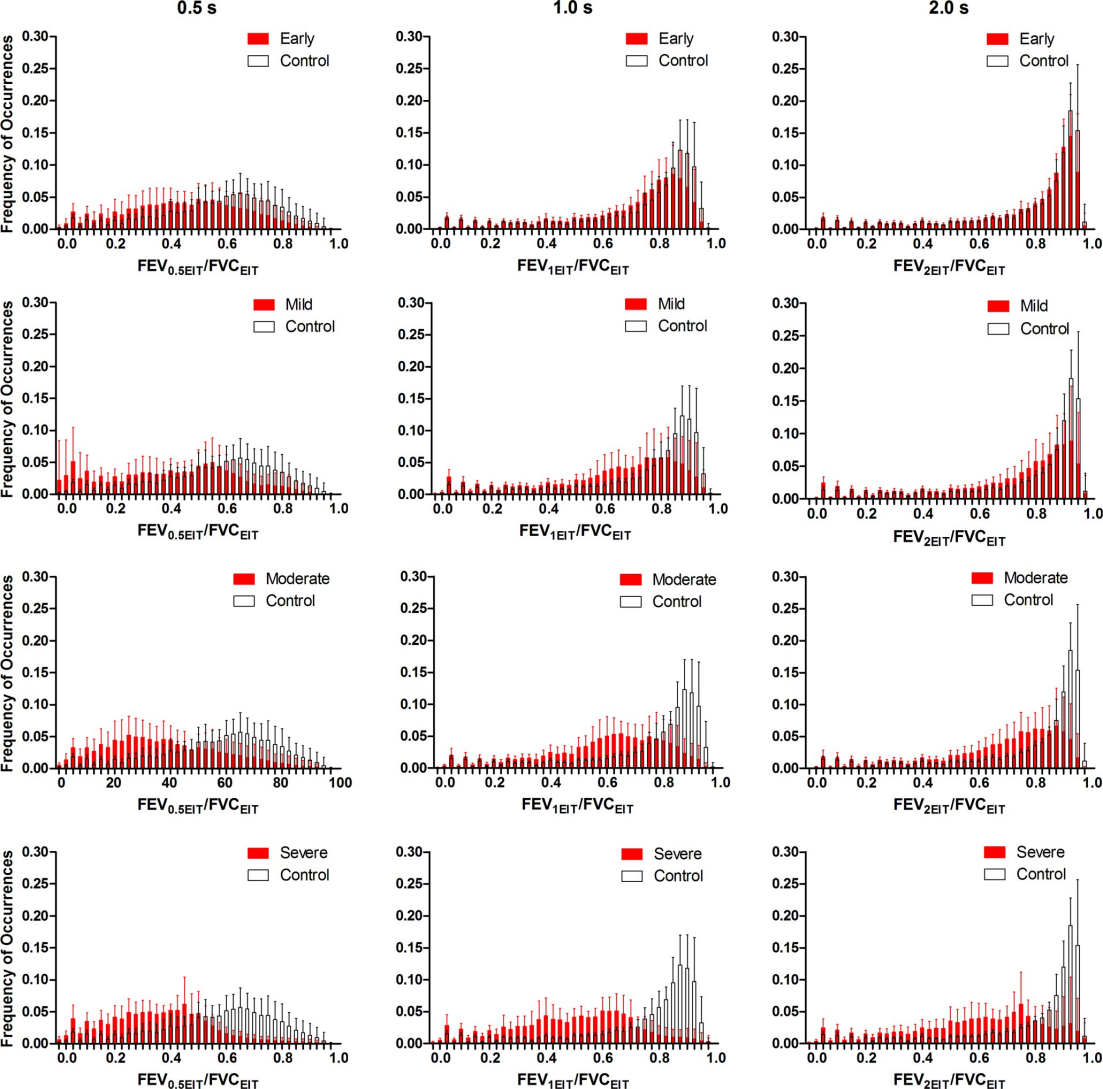
Histograms of pixel FEVt/FVC at different expiratory times. Data are expressed as mean ±SD. Figures show the histograms of FEVt/FVC at 0.5 s (left), 1 s (middle), and 2 s (right) in the groups with different degrees of obstruction (black columns) in comparison with healthy patients in the control group (red columns). FEV1, forced expiratory volume in 1 second; FVC, forced vital capacity.

Airway obstruction can occur in any part of the airway and lead to increased airway resistance. Meanwhile, COPD may also lead to the increase of lung compliance [32]. The increased airway resistance as well as the increased lung compliance prolong the expiratory time constants of the lung units [33], thus the lung units need a long time to fill and empty. The expiratory time constants of the regional lung units can be estimated approximately with the pixel FEV_t_/FVC value in the corresponding position of the fEIT image. Obviously, the expiratory time constant and FEV_t_/FVC are inversely related. Different time constants of the lung units lead to an inhomogeneous distribution of pixel FEV_t_/FVC determined by EIT. Therefore, the fEIT images of FEV_t_/FVC can reflect airway obstruction spatially and temporally. Fig 9 shows the influence of airway obstruction on the distribution of pixel FEVt/FVC at different degrees of obstruction and different expiratory times. Healthy subjects had the highest values of FEV_t_/FVC with a narrow peak. As the obstruction degree aggravated, the values got lower and shifted to the left with a broader peak. The results showed the capacity of EIT to reflect disease related flow limitation.

### Relationship between MI and the degree of obstruction

Lung compliance and airway resistance both affect the time constant of lung unit and lead to an inhomogeneous distribution of pixel FEV_1_/FVC. Gravity, interacting with posture, results in redistribution of gas in favor of the dependent lung and affects lung emptying capacity [29, 30]. Therefore, we validated the effectiveness of MI evaluating the degree of obstruction by evaluating body position related change of lung emptying capacity. The results verified that the proposed MI can be used to evaluate the inhomogeneity of regional ventilation caused by airway obstruction.

The degree of airway obstruction determines the expiratory time constants of lung units. Different expiratory time constants change the distribution of pixel FEV_1_/FVC. The peak of the histogram of pixel FEV_1_/FVC shows consistence with the degree of obstruction. So we chose the mode of pixel FEV1/FVC to characterize the degree of obstruction. The results of this study have validated the effectiveness of MI. In addition, we chose the mode of pixel FEV_1/_FVC because FEV_1_/FVC is an established value to characterize airflow limitation in PFT. Whether there exists another time t after the beginning of forced expiration, at which the histogram of pixel FEVt/FVC can better discriminate the degree of obstruction, remains to be discussed.

### Relationship between global and regional obstruction

The regional MI values indicated the presence of regional obstruction, and the degree of regional obstruction could be determined in the contour-map ROIs or in the four-quadrant ROIs. The degree of regional obstruction determined by EIT could be milder or severer than the global degree determined by spirometry. For example, subject D in Fig 6 was diagnosed as moderate degree of OVD in PFT, but the degrees of regional obstruction in the four-quadrant ROIs were determined as mild, severe, and moderate by EIT, respectively. The result verified the consistency and difference between the global obstruction and regional obstruction. Regional obstruction may exist even in healthy subjects with normal lung function, indicating the potential of EIT as an indicator of early airway obstruction. EIT may provide a new method to locate and evaluate regional obstruction in real time without risk from radiation, thus helping to estimate the progression and therapy effects of obstructive lung disease.

### Limitations and future research

1. This pilot study showed the effectiveness of MI to evaluate the global and regional degree of obstruction in patients with obstructive lung diseases. However, direct translation of the presented results (such as the cut-off values of MI) into clinical practice is limited. A larger patient cohort is still needed to affirm its effectiveness in the future.
2. Regional obstruction measured by EIT was not compared with an established imaging modality like computed tomography. This is a limitation of this study. Whereas, the feasibility of EIT to evaluate regional ventilation has been previously validated [34,35].

## Conclusions

The high temporal resolution of EIT makes it possible to analyze the change of regional distribution of ventilation over time and evaluate the degree of regional obstruction. This is an advantage of EIT comparing to spirometry. In the clinical setting, spirometry is the most common of the pulmonary function tests. It measures the volume and speed of air and is helpful in diagnosing and assessing the severity of lung function impairment. While the use of spirometry is limited for bedside monitoring. The application of EIT is still primarily done within a research context, but it may prove to be a useful bedside tool for monitoring and evaluating obstructive lung diseases in the future, which is helpful to analyze the regional pathological change and therapeutic effect in real-time. This is another advantage of EIT.

In summary, this study showed the potential of EIT to evaluate the degree of obstruction in patients with OVD on the global and regional level. EIT may become a new method in PFT to evaluate the progress and therapy effects of obstructive lung diseases.
